# Phosphodiesterase-induced cAMP degradation restricts hepatitis B virus infection

**DOI:** 10.1098/rstb.2018.0292

**Published:** 2019-04-08

**Authors:** Antonia Alexandra Evripioti, Ana Maria Ortega-Prieto, Jessica Katy Skelton, Quentin Bazot, Marcus Dorner

**Affiliations:** Section of Virology, Department of Medicine, Imperial College London, London, UK

**Keywords:** sodium taurocholate cotransporting polypeptide, phosphodiesterases, hepatitis B virus, viral entry

## Abstract

Hepatitis B virus (HBV) entry into hepatocytes is mediated via a high-affinity interaction between the preS1 glycoprotein and sodium/bile acid cotransporting polypeptide (NTCP). To date, *in vitro* model systems rely on high multiplicities of infection to achieve infection of cell lines overexpressing human NTCP. This study investigates a novel regulatory pathway for NTCP trafficking to the cell surface, induced by DMSO-mediated cellular differentiation. DMSO rapidly induces high cell surface expression of NTCP and results in increased susceptibility of cells to HBV infection. Additionally, DMSO treatment induces actin, as well as Tubulin reshaping within the cells. We show that direct disruption of the actin and Tubulin network directly enhances NTCP expression and the subsequent susceptibility of cells to HBV infection. DMSO induces these changes via alterations in the levels of cyclic (c)AMP, which participates in the observed actin rearrangements. Blocking of phosphodiesterases (PDEs), which degrade accumulated cAMP, had the same effect as DMSO differentiation and demonstrates that DMSO prevents phosphodiesterase-mediated cAMP degradation. This identifies adenylate cyclase as a novel target for blocking the entry of HBV via targeting the cell surface accumulation of NTCP.

This article is part of the theme issue ‘Silent cancer agents: multi-disciplinary modelling of human DNA oncoviruses’.

## 1. Introduction

Hepatitis B virus (HBV) results in over 257 million chronic infections worldwide and even though there is a protective vaccine available, nearly 4 million new infections arise every year. The virus belongs to the *Hepadnaviridae* family and has a partially double-stranded relaxed circular genome [1]. HBV is nowadays considered the leading cause of liver cancer, and unfortunately, there is no currently available therapy to cure the disease. Existing treatments rely primarily on nucleoside analogues (NUCs) and interferon alpha (IFN*α*), which work by suppressing the replication of the virus. These, however, are life-long and discontinuation uniformly results in viral relapse [2]. In addition, HBV can cause serious co-infections that are typically much harder to suppress in patients [3]. Therefore, new treatments are urgently required.

The sodium taurocholate cotransporting polypeptide (NTCP or SLC10A1) receptor is a glycosylated multiple transmembrane transporter predominantly expressed in the liver, with its polypeptide being exclusively localized to the basolateral membrane of hepatocytes, and is normally involved in the maintenance and circulation of bile acids in the enterohepatic area of the body [4,5].

In 2012, NTCP was reported as the long-sought entry receptor for HBV and hepatitis D virus (HDV) infection [6]. HBV binding and entry into hepatocytes is known to be facilitated with the help of its viral envelope glycoproteins, with the most critical site being its N-terminal myristoylated preS1 region [7]. The exact mechanisms of attachment and productive entrance into the host liver cells, however, are still enigmatic [8]. HBV entry into hepatocytes is separated into two distinct steps, where an initial low-affinity interaction with heparan sulfate proteoglycans (HSPGs), including the recently identified glypican 5 (GPC5) [9], on the hepatocyte surface, mediated by the preS1 region of the L (large) protein and the antigenic loop of the viral S (small) protein, is followed by the attachment of the virus to its high-affinity NTCP receptor [10]. This interaction then results in endocytosis of the HBV particles and further viral infection [11].

HBV research has been considerably impeded over the years by the lack of understanding of the molecular pathways of HBV entry into human liver cells. Current technologies, however, as well as the discovery of NTCP as the receptor for HBV infection, have opened up the field of HBV research and have allowed the establishment of *in vitro* systems for the study of HBV infection. One of the best examples is the exogenous expression of human NTCP in Huh7 and HepG2 cells that confers susceptibility to HBV and HDV infection to normally non-susceptible cells. Nevertheless, efficient infection of these cell lines requires the presence of both DMSO and polyethylene glycol (PEG) during inoculation, presumably partially by promoting virus attachment to the cell surface [12].

Previously, it was discovered that NTCP protein expression in HepG2–NTCP cells, despite being under the control of a cytomegalovirus immediate-early (CMV) promoter, was remarkably increased by the presence of DMSO [13]; this would partially explain the need for DMSO addition to increase the susceptibility of these cells to HBV infection [14]. However, so far, no extensive studies have been performed with the aim of dissecting the way by which DMSO is exerting this effect on the NTCP receptor, and in general, there appears to be an incomplete understanding of the vast effects of DMSO on different biological processes [15]. The effect of DMSO on NTCP appears to be an unusual process and probably much more complex than initially expected. DMSO, throughout history, has always been used mainly as a vehicle control and a solvent for water-insoluble reagents, and the only circumstances where DMSO has been used as a differentiation agent are in HepaRG cells [16] and hepatoma cell lines [17], as well as for stem cell differentiation. Some examples include differentiation of HL-60 cells to neutrophil-like cells [18], P19 cells into cardiac and skeletal muscle cells [19] and generally differentiation of human embryonic stem cells [20]. NTCP is the first reported case of DMSO having such an extensive effect on a receptor protein that lies beyond differentiation and polarization of cells for correct protein configuration and expression. Early studies of rat NTCP already suggest that NTCP expression is in general extensively regulated on a post-translational level through regulating cell surface expression via intracellular pools of cAMP and PKA [21,22]. However, data on human NTCP thus far have not implicated similar regulatory pathways.

Here, we describe the mechanistic regulation of NTCP by DMSO and its impact on the susceptibility of cells to HBV infection. DMSO exposure results in an accumulation of intracellular cyclic (c)AMP, which in turn acts as a cell polarization agent by restructuring cellular cortical actin and microtubules. This effect is at least partially mediated via phosphodiesterase (PDE)4, which is highly expressed in the liver. This mechanism may explain why DMSO addition is critical for achieving HBV susceptibility of cell lines overexpressing NTCP.

## 2. Results

### (a) Human NTCP cell surface expression and susceptibility to HBV infection are dependent on the presence of DMSO

**Figure 1.**
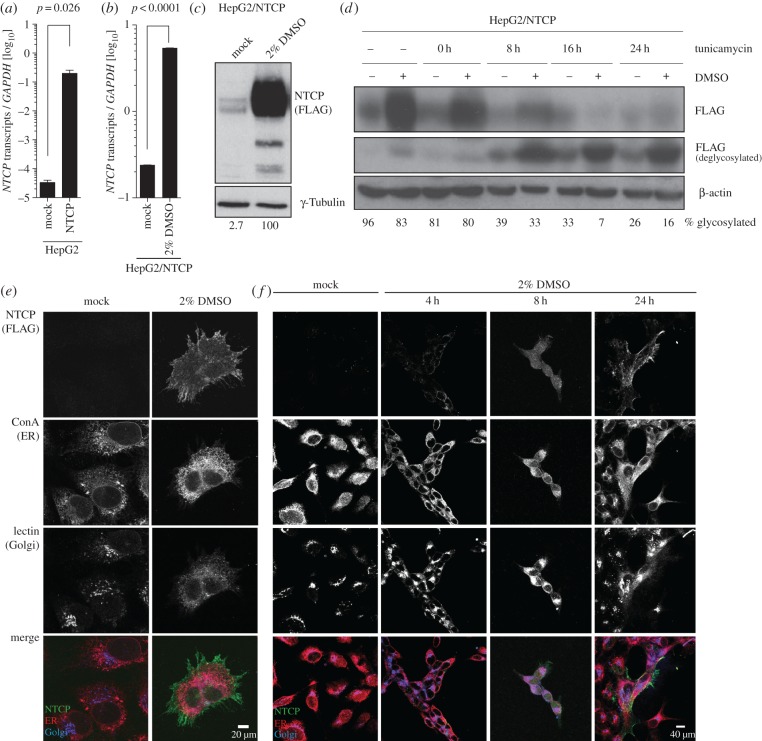
DMSO increases human NTCP levels during CMV promoter-driven overexpression, while it also increases endogenous cellular levels. (*a*) mRNA levels of NTCP in HepG2 cells following lentiviral transduction with a CMV promoter-driven FLAG-tagged NTCP construct. (*b*,*c*) NTCP (*b*) mRNA and (*c*) protein levels in HepG2 cells overexpressing NTCP, cultured either in the absence or presence of 2% DMSO for 24 h. (*d*) Glycosylation kinetic of NTCP in HepG2 cells following induction with 2% DMSO. Tunicamycin (5 µg ml^−1^) was added to the samples at the indicated time points to block all *de novo* glycosylation. (*e*,*f*) Immunofluorescence microscopy of NTCP following culture in the absence or presence of 2% DMSO for the indicated time frames. Cells were additionally stained with concanavalin A to visualize ER and lectin to visualize Golgi networks. Numbers under western blot lanes indicate densitometric analysis normalized to loading controls. Data shown are mean (s.d.) of three independent experiments.

Most cell lines do not express the HBV receptor NTCP. Thus, HepG2 cell lines were lentivirally transduced with a Flag-tagged NTCP driven by a CMV promoter. Stable cell lines were obtained after antibiotic selection and mRNA expression levels of NTCP were measured using quantitative (q)PCR, which showed that NTCP was overexpressed by 4-log10 in HepG2 cells (figure 1*a*). This expression was furthermore enhanced by culturing the cells in the presence of 2% DMSO (figure 1*b*). However, despite this apparent transcriptional overexpression, protein analysis of the former samples by western blot demonstrated that significantly less NTCP overexpression could be detected in HepG2 cells when cultured in complete DMEM in the absence of DMSO (figure 1*c*). Kinetic and dose-dependence analysis revealed that maximal NTCP expression is achieved following 15 h of exposure to 2% DMSO. Furthermore, removal of DMSO from the culture conditions resulted in a reduction of NTCP protein expression after 12 h (electronic supplementary material, figure S1). Since NTCP is highly glycosylated, we additionally determined the time required to produce functionally glycosylated NTCP upon exposure of cells to 2% DMSO. For this purpose, cells were exposed to 2% DMSO and Tunicamycin, an inhibitor of N-linked glycosylation that was added to the cultures at the indicated time points (figure 1*d*). This demonstrated that the first functionally glycosylated NTCP protein is detectable 8 h following exposure to DMSO. A similar kinetic was also determined by immunofluorescence microscopy, where NTCP is detectable on the ER of cells at 4 h post-DMSO exposure, and it passes through the Golgi network onto the cell surface between 8 and 24 h post-DMSO exposure (figure 1*e,f*). This effect is furthermore specific for NTCP, since using a similar CMV promoter-driven lentiviral construct did not exhibit DMSO sensitivity when expressing GFP as a control gene (electronic supplementary material, figure S2).

**Figure 2.**
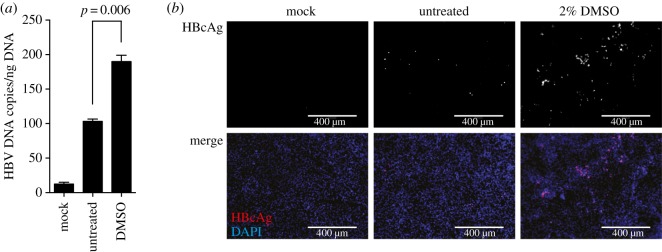
DMSO increases the susceptibility of HepG2–NTCP cells to HBV infection. (*a*) HBV DNA secretion and (*b*) HBcAg immunofluorescence staining of untreated HepG2–NTCP cells or cells cultured in the presence of 2% DMSO for 24 h following infection using 1000 GE/cell HBV. HBV DNA and HBcAg were determined 5 days post infection of cells. Data shown are mean (s.d.) of three independent experiments.

To determine the functional consequence of DMSO-induced NTCP expression, we performed HBV infections using a multiplicity of infection of 1000 HBV genome equivalents per cell in the absence or presence of DMSO differentiation for 24 h (figure 2*a*,*b*). Both, analysis of secreted HBV DNA (figure 2*a*) and immunofluorescence analysis of HBcAg-positive cells (figure 2*b*), revealed that 24 h exposure of HepG2–NTCP cells to 2% DMSO greatly enhances HBV susceptibility of these cells.

### (b) DMSO treatment reorganizes the cellular actin network, resulting in increased NTCP expression and HBV susceptibility

**Figure 3.**
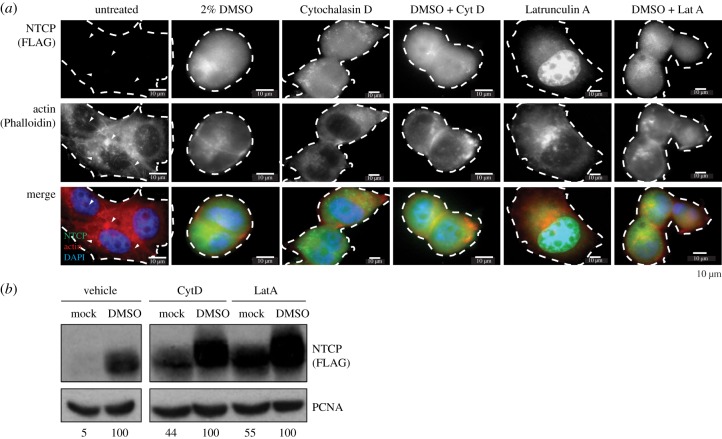
DMSO causes actin destabilization and interference with actin filaments results in NTCP expression. (*a*,*b*) Impact of 24 h treatment with DMSO, and/or 4 µg ml^−1^ Cytochalasin D or 0.1 µg ml^−1^ Latrunculin A, on actin filaments and NTCP expression in HepG2–NTCP cells as determined by (*a*) immunofluorescence microscopy, including staining with Phalloidin and NTCP, as well as (*b*) western blot for NTCP. Numbers under western blot lanes indicate densitometric analysis normalized to loading controls. Data shown are representative images of three independent experiments.

Despite the use of DMSO in cellular differentiation of hepatoma cell lines, little is known in regard to the molecular events resulting in the increase in HBV susceptibility of DMSO-differentiated HepG2–NTCP cells. In addition to inducing NTCP expression, DMSO also results in morphological changes to the cell, as well as loss of visible actin stress fibres (figure 3*a*). To evaluate whether this effect is independent of the observed increase in NTCP protein expression, we determined whether targeted disruptions of either the actin or microtubule network influence NTCP levels (figure 3*a*,*b*). Strikingly, targeted depolymerization of actin using either Cytochalasin D (CytoD—4 µg ml^−1^) or Latrunculin A (LatA—0.1 µg ml^−1^), in the absence of DMSO, resulted in the expression of NTCP at the cell surface (figure 3*a*). Comparative analysis using western blot furthermore demonstrated that the individual effect of Cytochalasin D and Latrunculin A was as pronounced as treatment of cells with 2% DMSO, even though combination with DMSO resulted in a synergistic effect on NTCP expression (figure 3*b*). Latrunculin A, which not only prevents actin polymerization but also enhances actin depolymerization, exhibited a stronger effect on the induction of NTCP cell surface expression. Since it is well established that microtubule regulation and actin filament maintenance are interlinked, coordinating cell motility, and both actin filaments and microtubules can regulate protractive and contractile forces, we further evaluated the impact of microtubule-modifying agents on NTCP expression. Thus, cells were treated with compounds disrupting microtubule dynamics, including Colchicine, demecolcine and Taxol. Even though, similar to disrupting actin fibres, microtubule disruption resulted in enhanced expression of NTCP, this was not as pronounced as compared to Cytochalasin D or Latrunculin A (electronic supplementary material, figure S3).

**Figure 4.**
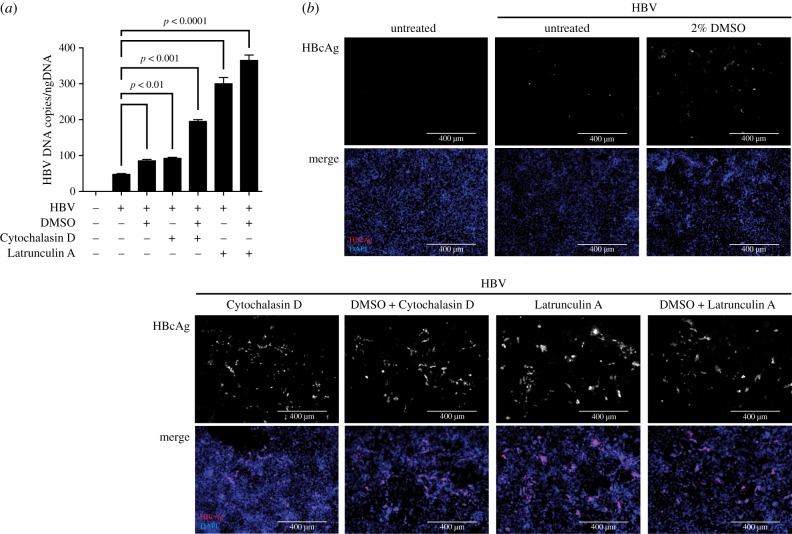
Disruption of actin filaments increases the susceptibility of HepG2–NTCP cells to HBV infection. (*a*) HBV DNA secretion and (*b*) HBcAg immunofluorescence staining of untreated HepG2–NTCP cells or cells cultured in the presence of 2% DMSO, and/or 4 µg ml^−1^ Cytochalasin D or 0.1 µg ml^−1^ Latrunculin A, for 24 h following infection using 1000 GE/cell HBV. HBV DNA and HBcAg were determined 5 days post infection of cells. Data shown are mean (s.d.) of three independent experiments.

To assess the consequence of Cytochalasin D and Latrunculin A treatment for the relative susceptibility to HBV infection, HepG2–NTCP cells were pre-treated with either 2% DMSO and/or Cytochalasin D or Latrunculin A for 24 h prior to infection with 1000 HBV genome equivalents per cell (figure 4). As evidenced by secretion of HBV DNA (figure 4*a*) and immunofluorescence staining of HBcAg (figure 4*b*), treatment of cells with either Cytochalasin D or Latrunculin A, in the absence of DMSO, rendered cells more susceptible to HBV infection compared to exposure to DMSO alone. Similar to NTCP protein expression, the susceptibility of cells was greatest when disrupting actin filaments with Latrunculin A, compared to Cytochalasin D. This demonstrates that in HepG2–NTCP cells, the actin network restricts HBV infection through blocking NTCP cell surface expression.

### (c) DMSO interferes with actin polymerization by increasing intracellular cAMP levels

**Figure 5.**
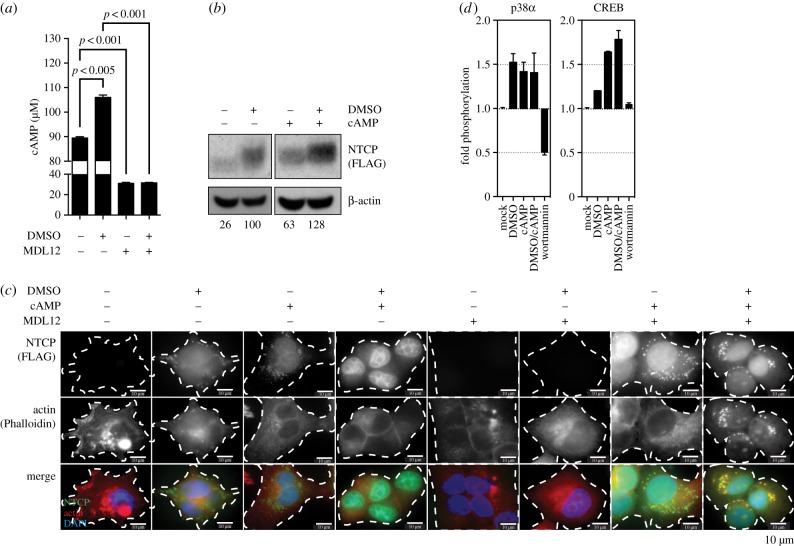
DMSO results in an increase in cAMP levels, which in turn increases cell surface human NTCP expression in a PI3 K/CREB-dependent manner. (*a*,*b*) Impact of DMSO, as well as MDL12—an inhibitor of adenylyl cyclase—on intracellular levels of cAMP. (*b*,*c*) Impact of DMSO or exogenously added cAMP on cellular NTCP levels as determined by (*b*) western blot and (*c*) immunofluorescence microscopy. (*d*) Activation of PI3 K signalling, as determined by phosphorylation analysis of p38*α* and CREB in the presence of 2% DMSO, exogenous cAMP or the p38 inhibitor Wortmannin, performed by phospho-kinase arrays. Numbers under western blot lanes indicate densitometric analysis normalized to loading controls. Data shown are mean (s.d.) of three independent experiments.

It has previously been documented that rat NTCP plasma membrane localization is dependent on the phosphorylation status of NTCP, which is cAMP-dependent [23]. Furthermore, cAMP has been shown to impact actin phosphorylation, thus contributing to active depolymerization of actin filaments [24]. Modulation of F-actin and cell mobility largely depend on the intracellular pool of cAMP, which regulates actin polymerization via protein kinase A, p38 mitogen-activated protein kinases and cAMP-responsive element binding protein (CREB) [25]. To assess whether DMSO resulted in increased cAMP we quantified intracellular cAMP levels following 24 h of contact of the agent with the cells, which demonstrated that DMSO increased cAMP within the cells (figure 5*a*). Blocking the biogenesis of cAMP using the specific adenylate cyclase inhibitor MDL12 greatly reduced intracellular cAMP levels and, most importantly, abolished the DMSO-induced accumulation of cAMP. Next, cells were treated with exogenous cAMP to assess whether this would, similarly to DMSO treatment, induce NTCP expression. Indeed, treatment of cells with 10 µM cAMP had the same effect on NTCP expression as DMSO stimulation (figure 5*b*). Immunofluorescence analysis confirmed that cAMP has a comparable effect to DMSO in inducing NTCP expression, which can be blocked by MDL12 and rescued by providing exogenous cAMP (figure 5*c*). Using phospho-kinase arrays to evaluate the phosphorylation of cAMP-responsive pathways, we furthermore determined that DMSO, as well as direct addition of exogenous cAMP, resulted in activation of p38*α* and CREB (figure 5*d*). This demonstrates that DMSO activates the production of cAMP, which in turn activates signal transduction pathways required for inducing actin depolymerization.

**Figure 6.**
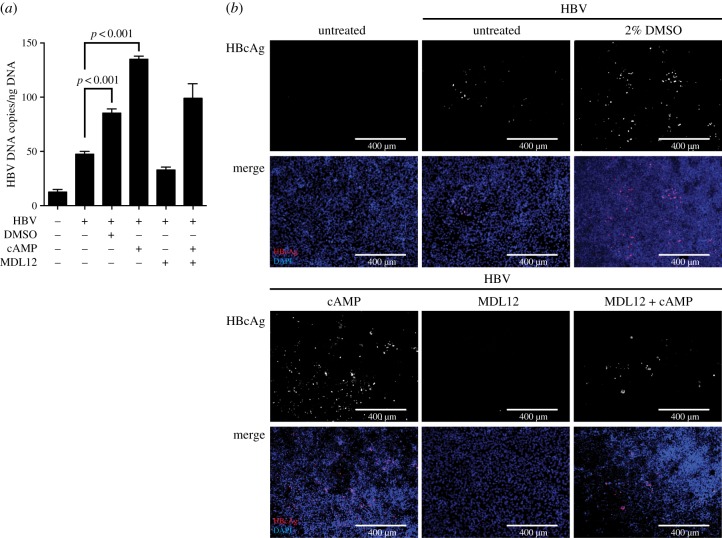
Exogenous cAMP increases the susceptibility of HepG2–NTCP cells to HBV infection. (*a*) HBV DNA secretion and (*b*) HBcAg immunofluorescence staining of untreated HepG2–NTCP cells or cells cultured in the presence of 2% DMSO, and/or 10 µM cAMP and MDL12, for 24 h following infection using 1000 GE/cell HBV. HBV DNA and HBcAg were determined 5 days post infection of cells. Data shown are mean (s.d.) of three independent experiments.

Comparable to the impact of cAMP on cellular NTCP expression, cells also exhibited increased susceptibility to HBV infection following the addition of exogenous cAMP (figure 6). Following infection with 1000 genome equivalents of HBV per cell, cAMP pre-treatment rendered cells more susceptible to HBV infection compared to DMSO treatment, as determined by secretion of HBV DNA and immunofluorescence staining of HBcAg (figure 6*a*,*b*). This shows that the DMSO-induced cAMP accumulation, as well as regulating downstream signalling, directly regulates the cells' susceptibility to HBV infection.

### (d) Phosphodiesterases degrading cAMP are responsible for restricting NTCP expression on cells

**Figure 7.**
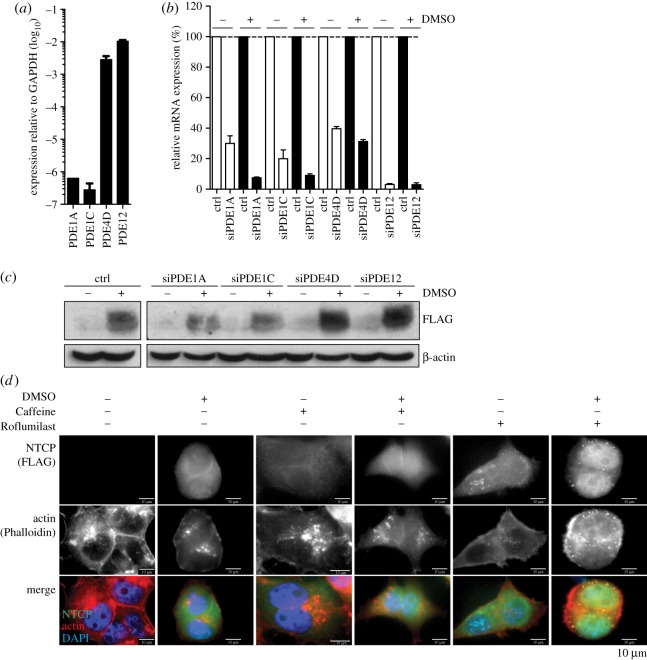
PDE4 and PDE12 restrict human NTCP expression in HepG2/NTCP cells. (*a*) mRNA expression of PDE1A, PDE1C, PDE4D and PDE12 in HepG2–NTCP cells. (*b*) Silencing efficiency of PDE1A, PDE1C, PDE4D and PDE12 in HepG2/NTCP cells, as determined by quantitative PCR. (*c*) Impact of lack of PDE1A, PDE1C, PDE4D and PDE12 expression on NTCP levels, either in the presence or absence of 2% DMSO treatment, as determined by western blot. (*d*) Immunofluorescence of NTCP and actin following treatment of HepG2–NTCP cells with DMSO, and/or Caffeine or Roflumilast. Data shown are mean (s.d.) of three independent experiments.

cAMP is degraded in cells through the action of phosphodiesterases, which comprise a total of 12 families, and which are characterized by their activity on cAMP or cGMP, as well as tissue expression. PDE4D and PDE12 are the most abundant phosphodiesterases in the liver acting specifically on cAMP [26]. To confirm their expression in HepG2–NTCP cells we quantified their mRNA expression, which demonstrated that particularly PDE4D and PDE12 are highly expressed in these cells as compared to PDE1A and PDE1C, which are only expressed at low levels (figure 7*a*). Next, to evaluate the role of phosphodiesterases in the DMSO-induced expression of NTCP, we used RNAi to specifically deplete PDE1A, PDE1C, PDE4D or PDE12 in HepG2–NTCP cells (figure 7*b*; electronic supplementary material, figure S4). Even though knock-down of neither phosphodiesterase itself resulted in NTCP expression, cells lacking PDE4D and PDE12 expressed significantly higher NTCP levels when exposed to DMSO (figure 7*c*). This was also independently confirmed by immunofluorescence microscopy following treatment of cells with the pan-phosphodiesterase inhibitor Caffeine, as well as with the PDE4D-specific inhibitor Roflumilast (figure 7*d*). Strikingly, pharmacological inhibition of phosphodiesterases, specifically PDE4D, using small-molecule inhibitors resulted in the induction of NTCP expression, even in the absence of DMSO treatment, in a dose-dependent manner (electronic supplementary material, figure S5). This shows that DMSO purposely inhibits cellular phosphodiesterases in HepG2–NTCP cells, preventing the degradation of cAMP.

**Figure 8.**
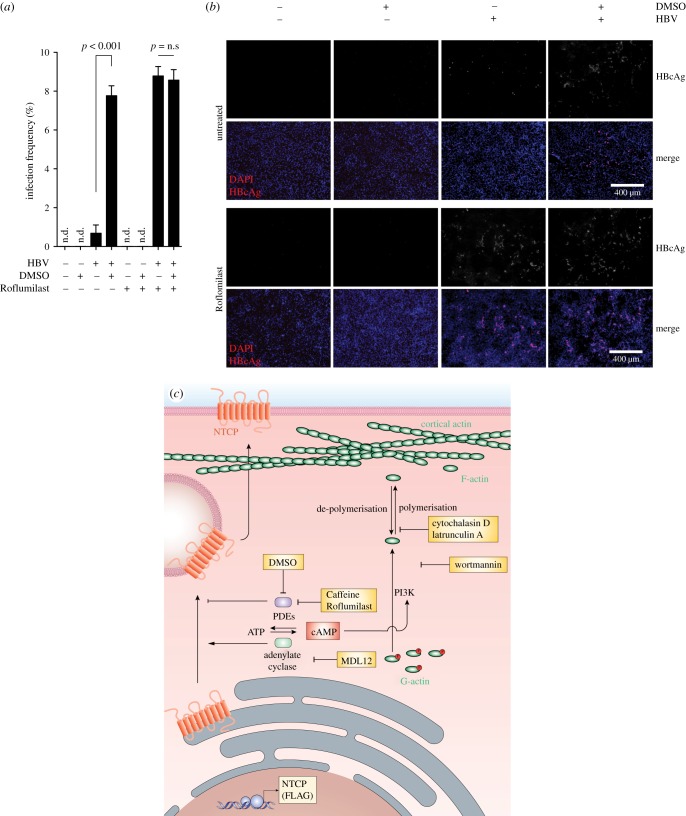
Inhibition of PDE4D increases the susceptibility of HepG2–NTCP cells to HBV infection. (*a*) HBcAg flow cytometry and (*b*) HBcAg immunofluorescence staining of untreated HepG2–NTCP cells or cells cultured in the presence of 2% DMSO, and/or 20 nM Roflumilast, for 24 h following infection using 1000 GE/cell HBV. HBV DNA and HBcAg were determined 5 days post infection of cells. Data shown are mean (s.d.) of three independent experiments. (*c*) Schematic pathway of DMSO-induced cell surface expression of NTCP.

To assess whether this DMSO-induced phosphodiesterase inhibition translates to enhanced susceptibility of cells to HBV infection, we treated HepG2–NTCP cells with DMSO and/or Roflumilast for 24 h before infection using 1000 genome equivalents of HBV per cell. As determined by immunofluorescence microscopy and flow cytometry of HBcAg-positive cells, the pharmacological inhibition of PDE4D increased the susceptibility of HepG2–NTCP cells approximately 10-fold, similar to the increase observed when pre-treating cells with DMSO (figure 8*a*,*b*). Strikingly, no synergistic effect was observed when combining DMSO with Roflumilast treatment, indicating that the infection-enhancing effect of DMSO is primarily exerted via the inhibition of PDE4D. However, modulation of intracellular cAMP levels by inhibition of PDE4D, adenylate cyclase or disruption of actin filaments still retained the dependency of HBV infection on NTCP, indicating that no alternative entry pathways exist in these cells (electronic supplementary material, figure S6).

## 3. Discussion

In the present study, we show how human NTCP expression is tightly regulated by the presence of DMSO in cell culture media, and how this affects the susceptibility of cells to HBV infection. While this phenomenon was previously described [27], we specifically demonstrate how the presence of DMSO enhances the expression of NTCP on a transcriptional and a translation level, while it also results in more NTCP being translocated to the cell surface. The effect of DMSO on NTCP appears to be a very precisely orchestrated procedure, which is both time- and dose-dependent, while it is specific to NTCP as other cellular proteins are not necessarily affected in the same manner. Additionally, DMSO directly affects NTCP and not the CMV promoter driving NTCP expression. Removing DMSO from the cell culture media can easily reverse this effect. In the absence of DMSO, the levels of NTCP expression quickly diminish, even though NTCP protein is definitely more stable than the mRNA. In addition to resulting in elevated cell surface expression of NTCP, DMSO furthermore alters the cellular cytoskeleton, resulting in the rearrangement of the actin and microtubule microstructures. This may be an indirect effect caused by the induction of cell stress; however, this effect directly coincides with an elevation of intracellular cAMP levels, which have previously been shown to play an important role in phosphorylation of actin [28–30]. Furthermore, disruption of actin filaments and microtubules directly results in elevation of cell surface NTCP in the absence of cAMP elevation, suggesting this to be among the final steps in facilitating NTCP trafficking to the plasma membrane.

The elevation of cAMP levels furthermore results in the initiation of a PI3 K-dependent signal transduction pathway, whose effect on NTCP cell surface expression can be counteracted by either blocking the PI3 K signal transduction through Wortmannin or inhibiting the *de novo* production of cAMP via targeting of adenylyl cyclase. This mechanism is clearly centred on cAMP, since the addition of exogenous cAMP to cells, in which adenylyl cyclase has been blocked, rescues this effect. Caffeine, which is a broad phosphodiesterase inhibitor, had the opposite effect and thus directly implicates PDE in restricting NTCP cell surface expression and the subsequent susceptibility of cells to HBV infection. Based on substrate specificity and tissue-expression levels, we narrow down the effect on PDE4, which directly controls NTCP cell surface expression levels and HBV susceptibility. Since pharmacological inhibition of PDE4 activity, by Roflumilast, completely abolishes the effect of DMSO on the susceptibility of HepG2–NTCP cells to HBV infection, we hypothesize that DMSO-mediated inhibition of PDE4 is the main mechanism by which DMSO increases the susceptibility of cells to HBV infection.

A similar effect on NTCP expression, as seen when inhibiting PDE4D, was also seen when inhibiting another liver-specific PDE, PDE12. However, currently there are no available small-molecule PDE12 inhibitors, so in the future, when one becomes available, it will be interesting to further investigate the effect of its inhibition on NTCP cell surface expression and susceptibility of cells to HBV infection, while also comparing it to the effect of Roflumilast.

Cellular phosphodiesterases, which are essential for forming second messenger molecules, including cAMP and cGMP, are usually characterized by their unique tissue-expression patterns [31]. Both cAMP and cGMP have previously been implicated in the regulation of ion channels, but the regulation of NTCP cell surface expression may be an additional function that might be regulated by liver-specific PDE. The engagement of PI3 K signalling via cAMP has already been demonstrated to be of importance for the translocation of rat NTCP to the cell surface, which involves the cAMP-initiated de-phosphorylation of NTCP [32]. However, the herein-described mechanism for NTCP post-translational regulation reveals a thus far unknown role for PDE-derived cAMP in the regulation of human NTCP plasma membrane transport (figure 8*c*).

The identification of NTCP as the *bona fide* receptor for HBV has sparked the development of novel entry inhibitors based on peptidomimetics of the HBV preS1 glycoprotein. These block HBV attachment to NTCP via a competitive interaction. Targeting the cAMP-mediated transport of NTCP to the cell surface may present a suitable alternative to restricting HBV access to its receptor and thus preventing infection events.

Interestingly, most currently used HepG2–NTCP cell lines for the study of HBV infection are monoclonal cell lines, which were selected on the basis of facilitating HBV infection upon treatment with DMSO. This may have resulted in the selection of cell clones with low baseline expression of distinct PDE. However, infection of these cell lines with HBV still requires very high multiplicities of infection and the presence of PEG during infection, suggesting the presence of additional receptors/co-receptors or host factors required for events essential to ensure viral persistence downstream of receptor-mediated entry.

## 4. Material and methods

### (a) Chemicals, reagents and antibodies

Paraformaldehyde (PFA), Triton^™^ X-100, Tunicamycin, Cytochalasin D, Latrunculin A, Colchicine, Demecolchine, Taxol, cAMP, Wortmannin, MDL12, Caffeine and Roflumilast were obtained from Sigma-Aldrich. DMSO was from VWR International. For detection of the NTCP protein, an anti-FLAG antibody (DYKDDDDK Epitope Tag antibody; ThermoFisher Scientific) was used, while for the detection of HBV core particles, an anti-HBcAg was ordered from Dako, Agilent Technologies Antibodies against β-actin and γ-Tubulin were from Sigma-Aldrich, while PCNA was obtained from Abcam. Small interference RNAs (siRNAs) were ordered from ThermoFisher Scientific. cAMP direct immunoassay kit was purchased from Abcam. Human phospho-kinase arrays were performed according to the manufacturer's instructions (R&D Systems).

### (b) Cell culture

HepG2 (human liver hepatoma) cells were purchased from ATCC. HepDE19 cells were provided by Haitao Guo (Indiana University). All cells were maintained in Dulbecco's modified Eagle's medium (DMEM; Gibco) further supplemented with 10% fetal bovine serum (FBS; Gibco), referred to as complete medium. HepDE19 cells were maintained in DMEM/F12 medium supplemented with 10% FBS, 500 µg ml^−1^ G418 and 1 µg ml^−1^ Tetracycline (Sigma-Aldrich). Cells were grown in a 5% CO_2_ atmosphere in a humidified incubator set at 37°C.

### (c) NTCP overexpression

Human NTCP cDNA cloned into a pReceiver lentiviral vector, driven by a CMV promoter and encoding a C-terminal FLAG-tag, was purchased from GeneCopoeia [EX-C0391-LV158]. Lentiviral pseudoparticles expressing human NTCP were prepared as previously described [33]. Briefly, HEK293T cells were transfected with three plasmids expressing the human immunodeficiency (HIV) gag/pol proteins, the vesicular stomatitis virus glycoprotein (VSVg) and the NTCP construct. Supernatants were collected at 48 and 72 h, filtered and supplemented with 20 mM Hepes (Gibco) and 4 µg/ml Polybrene (Sigma-Aldrich) before further use. For the creation of stable cell lines, HepG2 cells were transduced with the lentiviral pseudoparticles and the medium was changed 48 h post transduction to select stable HepG2–NTCP cells with G418 (Geneticin 400 µg ml^−1^; Sigma-Aldrich).

### (d) Real-time PCR

Total RNA was extracted from cells using the RNeasy^®^ Mini kit (Qiagen). Complementary DNA (cDNA) was synthesized using a high-capacity cDNA reverse transcription kit (ThermoFisher Scientific). Quantitative real-time polymerase chain reaction (qRT-PCR) was performed on a QuantStudio^™^ 7 Flex Real-Time PCR system using the Taqman assay system (ThermoFisher Scientific). Taqman^®^ Gene Expression assays against NTCP (human NTCP Hs00161820_m1) and PDEs 1A, 1C, 4D and 12 (Hs00897273_m1, Hs01095682_m1, Hs01579625_m1 and Hs00698272_m1, respectively) were from ThermoFisher Scientific. Expression levels were normalized to GAPDH reference gene (Hs02786624_g1; ThermoFisher Scientific) and were specifically analysed using the 2ΔΔC_T_ formula on the Prism 5 computer software (GraphPad).

### (e) Immunoblotting

For protein analysis, whole-cell lysates were obtained by lysing the cells in 1x radioimmunoprecipitation assay (RIPA) buffer. The samples were rapidly vortexed and kept on ice for 30 min prior to centrifugation for the removal of insoluble material. Proteins were separated on a 10% polyacrylamide gel, transferred to nitrocellulose membranes and probed with suitable antibodies.

### (f) Immunofluorescence

Cells were grown to approximately 80% confluence on coverslips; they were washed with phosphate-buffered saline (PBS), fixed with 4% PFA/PBS for 20 min at room temperature, washed three-times, permeabilized with 0.1% Triton X-100/PBS for 5 min at room temperature. After washing with PBS, nonspecific binding of the antibodies was blocked with 10% FBS/PBS for 30 min at room temperature. They were then stained for 1 h at room temperature with the rabbit anti-FLAG antibody diluted in 5% FBS/PBS, followed by an anti-rabbit AlexaFluor^®^ 488 (ThermoFisher Scientific) for one hour at room temperature. In the case of HBcAg detection, an AlexaFluor^®^ 594 conjugated secondary antibody was used for visualization. For specific staining of the ER and the Golgi, AlexaFluor^®^ 594 Concanavalin A and AlexaFluor^®^ 647 Lectin HPA (Helix pomatia agglutinin; ThermoFisher Scientific) antibodies were used respectively to stain the cells for a further hour at room temperature. For specific staining of actin and Tubulin, AlexaFluor^®^ 647 Phalloidin and a mouse a-Tubulin (Sigma-Aldrich) were used, respectively. For Tubulin, a secondary anti-mouse AlexaFluor^®^ 594 antibody was used for visualization purposes. Images were taken on an Axiovert 135 TV (Zeiss) fluorescent microscope or an EVOS FL Auto Cell Imaging System (ThermoFisher Scientific), as well as a confocal microscope (LSM Pascal, Zeiss).

### (g) Flow Cytometry

Cells were fixed with 4% PFA/PBS in suspension. The cells were either permeabilized with 0.1% Triton X-100/PBS or were not permeabilized. Unspecific binding of antibodies was blocked as previously described, followed by staining of the cells with a PE-conjugated anti-FLAG (L5 clone; BioLegend) antibody for 1 h at room temperature. The residual antibody was then washed away from the cells and the cells were immediately run on a BD LSR II system (BD Biosciences). Data were analysed using the FlowJo v7.61 software (Treestar).

### (h) HBV infections

Infectious HBV was generated from HepG2.2.15 cells (kindly provided by Peter Karayiannis, University of Nicosia) or HepDE19 cells. For infection experiments, cells were seeded at 80% confluence on collagen-coated plates. Cells were maintained in complete medium, in the presence or absence of 2% DMSO, for 24 h prior to infection. Infections, in the presence of 2% DMSO and 4% PEG 8000 (Sigma-Aldrich), were performed overnight (16 h). Total DNA was extracted from the collected cells using a DNeasy^®^ Blood & Tissue kit (Qiagen). HBV DNA was detected by qPCR using specific HBV PCR primers as previously described [34]. qPCR was performed on a QuantStudio^™^ 7 Flex Real-Time PCR system.

### (i) Statistics

Statistical analyses were performed using the GraphPad Prism Software. Statistics were calculated using one-way ANOVA analysis. *p*-Values below 0.05 were considered statistically significant.

## Supplementary Material

Supplementary figure 1

## Supplementary Material

Supplementary figure 2

## Supplementary Material

Supplementary figure 3

## Supplementary Material

Supplementary figure 4

## Supplementary Material

Supplementary figure 5

## Supplementary Material

Supplementary figure 6

## Data accessibility

This article has no additional data.
