# Supplementary material for: Phosphodiesterase-induced cAMP degradation restricts hepatitis B virus infection
Source: Philos Trans R Soc Lond B Biol Sci. 2019 Apr 8;374(1773):20180292. doi: 10.1098/rstb.2018.0292 (PMC6501904; doi:10.1098/rstb.2018.0292)

Figure S1. Kinetic and dose-dependence of NTCP expression by DMSO. (a) Western blot analysis of NTCP protein expression in HepG2-NTCP cells following 24 hours of culture in the absence or presence of DMSO at the indicated concentrations. (b) Western blot kinetic analysis of NTCP protein expression following culture of HepG2-NTCP cells in the absence or presence of 2% DMSO at the indicated time points. (c) Kinetic of NTCP protein expression in HepG2-NTCP cells following initial culture in medium containing 2% DMSO for 24 hours subsequently followed by withdrawal of DMSO for the indicated time points. Data shown are representative examples of three independent experiments.

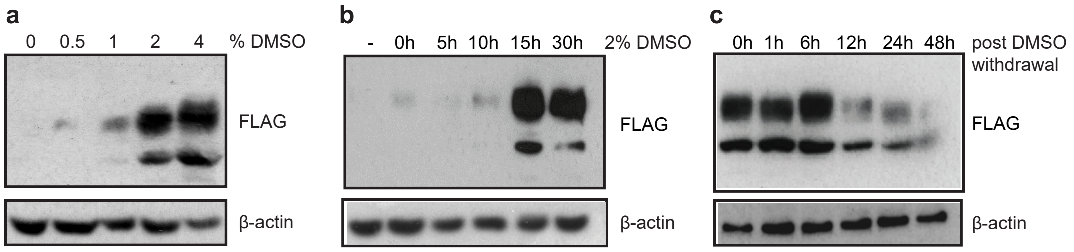

Supplement: Supplementary figure 1 [file rstb20180292supp1.pdf]
