# Supplementary material for: Phosphodiesterase-induced cAMP degradation restricts hepatitis B virus infection
Source: Philos Trans R Soc Lond B Biol Sci. 2019 Apr 8;374(1773):20180292. doi: 10.1098/rstb.2018.0292 (PMC6501904; doi:10.1098/rstb.2018.0292)

Figure S2. Specificity of the effect of DMSO on NTCP expression. (a-c) Effect of 2% DMSO on GFP expression in HepG2 cells through transfection using a CMV promoter-driven GFP construct 24 hours following DMSO treatment as determined by (a, b) immunofluorescence microscopy and (c) western blot of GFP. Data shown are representative examples of three independent experiments.

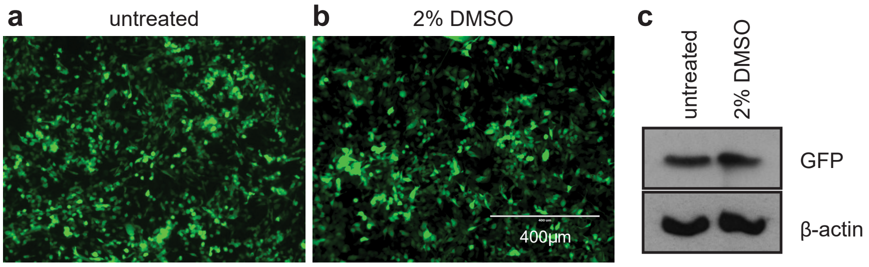

Supplement: Supplementary figure 2 [file rstb20180292supp2.pdf]
