# Supplementary material for: Phosphodiesterase-induced cAMP degradation restricts hepatitis B virus infection
Source: Philos Trans R Soc Lond B Biol Sci. 2019 Apr 8;374(1773):20180292. doi: 10.1098/rstb.2018.0292 (PMC6501904; doi:10.1098/rstb.2018.0292)

Figure S3. Microtubule destabilisation results in NTCP expression. (a,b) Impact of 24 hours treatment with DMSO, and/or Colchicine, Demecolchine or Taxol, on NTCP expression in HepG2-NTCP cells, as determined by (a) immunofluorescence microscopy, including staining with Phalloidin and NTCP, as well as (b) western blot for NTCP. Data shown are representative images of three independent experiments.

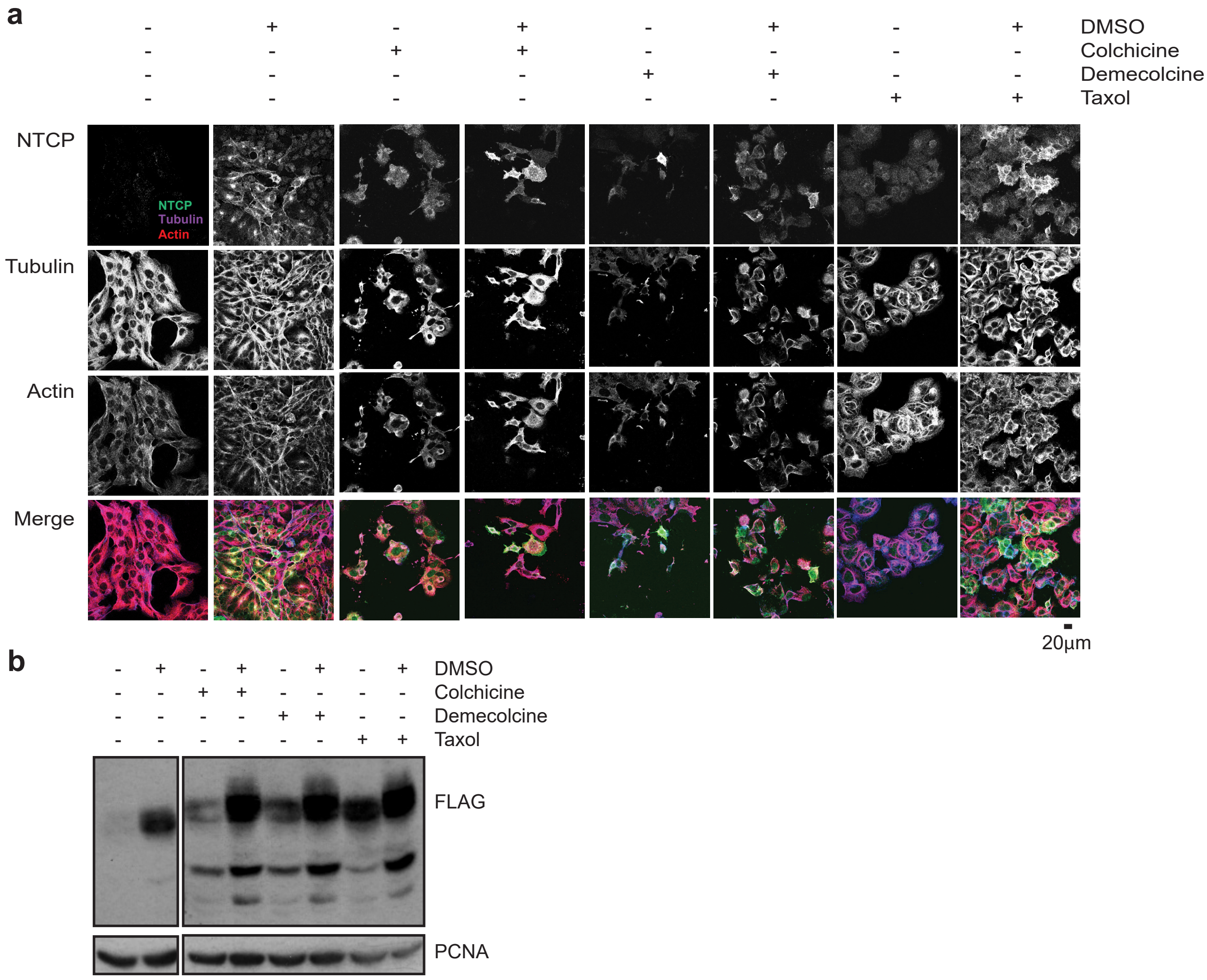

Supplement: Supplementary figure 3 [file rstb20180292supp3.pdf]
