# Supplementary material for: Phosphodiesterase-induced cAMP degradation restricts hepatitis B virus infection
Source: Philos Trans R Soc Lond B Biol Sci. 2019 Apr 8;374(1773):20180292. doi: 10.1098/rstb.2018.0292 (PMC6501904; doi:10.1098/rstb.2018.0292)

Figure S4. Silencing efficiency of PDE4D. Western blot analysis of HepG2-NTCP cells transfected with either control or PDE4D-targeting siRNA. Data shown are representative images of three independent experiments.

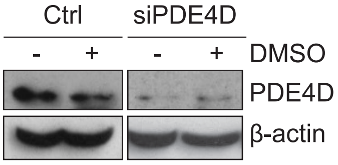

Supplement: Supplementary figure 4 [file rstb20180292supp4.pdf]
