# Supplementary material for: Phosphodiesterase-induced cAMP degradation restricts hepatitis B virus infection
Source: Philos Trans R Soc Lond B Biol Sci. 2019 Apr 8;374(1773):20180292. doi: 10.1098/rstb.2018.0292 (PMC6501904; doi:10.1098/rstb.2018.0292)

**Figure S5. Dose dependence of NTCP expression upon PDE4D inhibition.** Immunofluorescence analysis of NTCP expression on HepG2-NTCP cells 24 hours after inhibition of PDE4D using the indicated concentrations of Roflumilast. Data shown are representative images of three independent experiments.

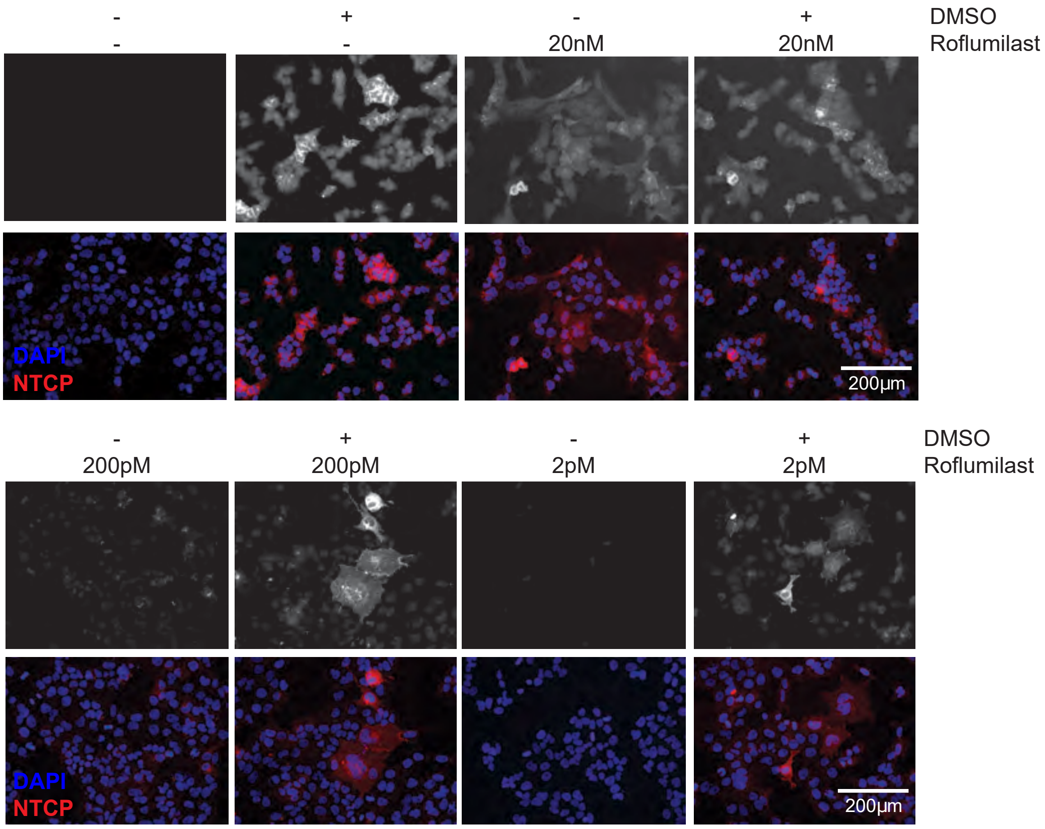

Supplement: Supplementary figure 5 [file rstb20180292supp5.pdf]
