# Supplementary material for: Phosphodiesterase-induced cAMP degradation restricts hepatitis B virus infection
Source: Philos Trans R Soc Lond B Biol Sci. 2019 Apr 8;374(1773):20180292. doi: 10.1098/rstb.2018.0292 (PMC6501904; doi:10.1098/rstb.2018.0292)

Figure S6. HBV infection of HepG2/NTCP cells depends on NTCP interaction. Evaluation of HBV susceptibility of HepG2/NTCP cells either left untreated or treated for 24 hours with 2% DMSO, Cytochalasin D, Wortmannin, cAMP, MDL12, MDL12 and cAMP or Roflumilast. Cells were either directly infected with HBV or were treated for 1 hour with 1μM myristroylated preS1 (aa1-49) prior to HBV infection to block binding of HBV to NTCP. Myristoylated preS1 was maintained for the entire infection and infection frequency was assessed 5 days post infection by immunofluorescence analysis of HBcAg (red) and DAPI (blue). Data shown are representative images of three independent experiments.

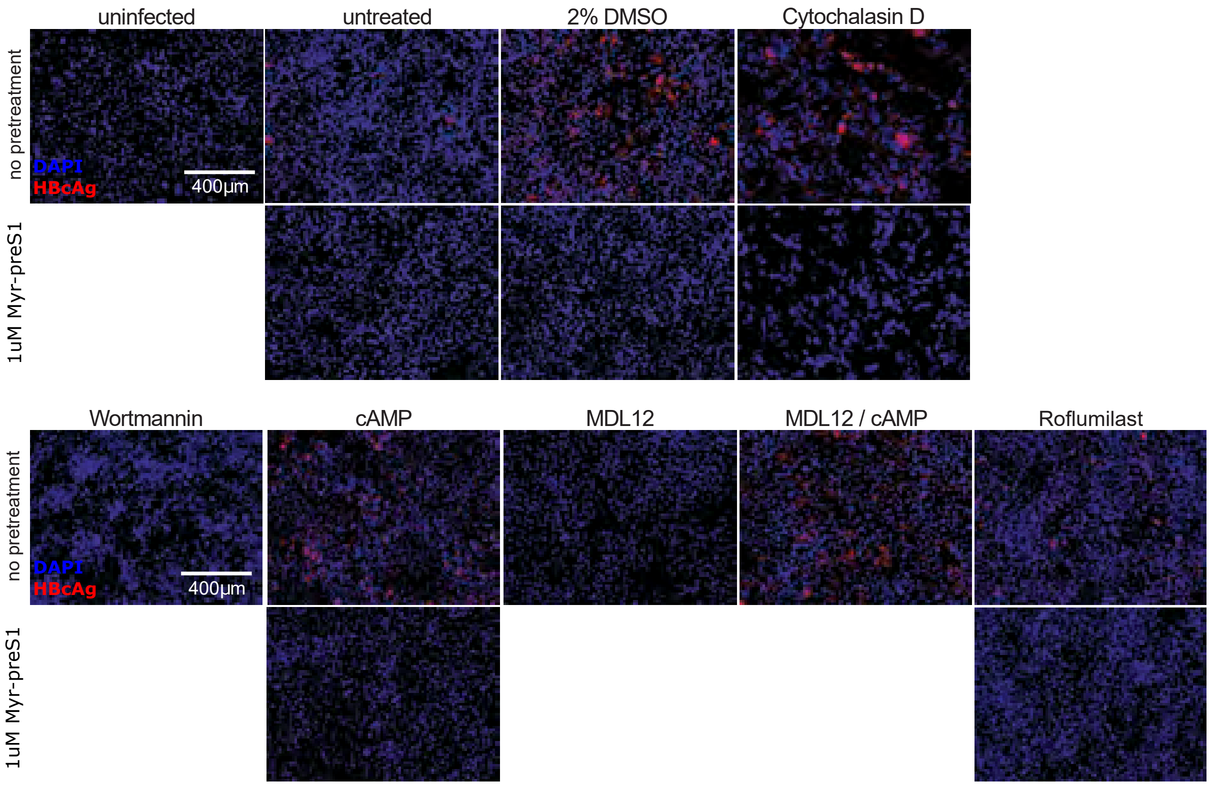

Supplement: Supplementary figure 6 [file rstb20180292supp6.pdf]
